# PERCEIVED AND PERFORMANCE FATIGABILITY IN UNDERSTANDING HEALTHY AGING: STUDY OF MUSCLE, MOBILITY AND AGING (SOMMA)

**DOI:** 10.1093/geroni/igae098.0235

**Published:** 2024-12-31

**Authors:** Nancy Glynn, Peggy Cawthon, Jennifer Schrack

**Affiliations:** Chair; Co-Chair; Discussant

## Abstract

Characterizing fatigability, the quantification of an individual’s susceptibility to fatigue in the context of standardized physical task(s), is a more sensitive approach to assess the presence and severity of fatigue, a critical prodromal trait of chronic and acute health conditions. We have validated measures of perceived physical fatigability (Pittsburgh Fatigability Scale, PFS) and performance fatigability (Performance Deterioration [PD, measured during a fast-paced 400m walk by calculating gait speed of lap2-lap9/lap2*100] and the Pittsburgh Performance Fatigability Index [PPFI, measured during a usual-paced 400m by using wrist-worn raw accelerometry data to quantify percent cadence decline). Our symposium will capitalize on the extensive phenotype data collected at baseline in SOMMA (N=879, mean age 76.3±5.0 years; walking speed 1.04±0.20 m/s; 59.2% women; 85% white), to further elucidate the correlates and consequences associated with greater perceived and/or performance fatigability in order to inform potential interventions to promote healthy aging. First, Ms. Reagan Garcia will examine whether time spent in activPAL measured sedentary behaviors are related to higher perceived physical fatigability (PFS). Next, Dr. Kyle Moored will share findings on the role of maximal treadmill cardiorespiratory fitness (VO2peak) and PFS on life-space mobility and whether these associations are moderated by perceived neighborhood factors. Dr. Caterina Rosano will explore the association between nigrostriatal dopaminergic integrity and performance fatigability (PD). Lastly, Ms. Emma Gay will be comparing skeletal muscle energetics and walking energetics profiles of those with lower PFS/lower PPFI vs. higher PFS/higher PPFI. Discussant, Dr. Jennifer Schrack will offer insights for new directions for fatigability research.

